# Schmallenberg Virus Pathogenesis, Tropism and Interaction with the Innate Immune System of the Host

**DOI:** 10.1371/journal.ppat.1003133

**Published:** 2013-01-10

**Authors:** Mariana Varela, Esther Schnettler, Marco Caporale, Claudio Murgia, Gerald Barry, Melanie McFarlane, Eva McGregor, Ilaria M. Piras, Andrew Shaw, Catherine Lamm, Anna Janowicz, Martin Beer, Mandy Glass, Vanessa Herder, Kerstin Hahn, Wolfgang Baumgärtner, Alain Kohl, Massimo Palmarini

**Affiliations:** 1 MRC Centre for Virus Research, Institute of Infection, Immunity and Inflammation, College of Medical, Veterinary and Life Sciences, University of Glasgow, Glasgow, United Kingdom; 2 Istituto G. Caporale, Teramo, Italy; 3 Dipartimento di Medicina Veterinaria, Università degli Studi di Sassari, Sassari, Italy; 4 Institute of Diagnostic Virology, Friedrich-Loeffler-Institut, Greifswald-Insel Riems, Germany; 5 Department of Pathology and Center of Systems Neuroscience, University of Veterinary Medicine, Hannover, Germany; Institute for Virology, Germany

## Abstract

Schmallenberg virus (SBV) is an emerging orthobunyavirus of ruminants associated with outbreaks of congenital malformations in aborted and stillborn animals. Since its discovery in November 2011, SBV has spread very rapidly to many European countries. Here, we developed molecular and serological tools, and an experimental *in vivo* model as a platform to study SBV pathogenesis, tropism and virus-host cell interactions. Using a synthetic biology approach, we developed a reverse genetics system for the rapid rescue and genetic manipulation of SBV. We showed that SBV has a wide tropism in cell culture and “synthetic” SBV replicates *in vitro* as efficiently as wild type virus. We developed an experimental mouse model to study SBV infection and showed that this virus replicates abundantly in neurons where it causes cerebral malacia and vacuolation of the cerebral cortex. These virus-induced acute lesions are useful in understanding the progression from vacuolation to porencephaly and extensive tissue destruction, often observed in aborted lambs and calves in naturally occurring Schmallenberg cases. Indeed, we detected high levels of SBV antigens in the neurons of the gray matter of brain and spinal cord of naturally affected lambs and calves, suggesting that muscular hypoplasia observed in SBV-infected lambs is mostly secondary to central nervous system damage. Finally, we investigated the molecular determinants of SBV virulence. Interestingly, we found a biological SBV clone that after passage in cell culture displays increased virulence in mice. We also found that a SBV deletion mutant of the non-structural NSs protein (SBVΔNSs) is less virulent in mice than wild type SBV. Attenuation of SBV virulence depends on the inability of SBVΔNSs to block IFN synthesis in virus infected cells. In conclusion, this work provides a useful experimental framework to study the biology and pathogenesis of SBV.

## Author Summary

Schmallenberg virus (SBV) was discovered in Germany (near the town of Schmallenberg) in November 2011 and since then has been found to be the cause of malformations and stillbirths in ruminants. SBV has spread very rapidly to many European countries including the Netherlands, Belgium, France and the United Kingdom. Very little is known about the biological properties of this virus and there is no vaccine available. In this study (i) we developed an approach (called reverse genetics) that allows the recovery of “synthetic” SBV under laboratory conditions; (ii) we developed a mouse model of infection for SBV; (iii) we showed that SBV replicates in neurons of experimentally infected mice similar to naturally infected lambs and calves; (iv) we developed viral mutants that are not as pathogenic as the original virus due to the inability to counteract the host cell defenses; and v) we identified mutations that are associated with increased virulence. This work provides the experimental tools to understand how this newly emerged virus causes disease in ruminants. In addition, it will now be possible to manipulate the SBV genome in order to develop highly effective vaccines.

## Introduction

Approximately 30 percent of all infectious diseases that emerged between 1990 and 2000 were caused by arthropod-borne viruses (arbovirus) [1]. This is probably the result of a combination of factors including a dramatic increase in travelling and commercial exchanges, climate and ecological changes and increased livestock production. In addition, changes in trading and commercial policies have created optimal conditions for the movement of infected vertebrate hosts and invertebrate vectors over wide geographical areas.

Several European countries are currently experiencing the emergence of a previously uncharacterized arbovirus of domesticated ruminants, Schmallenberg virus (SBV) [2], [3]. SBV infection causes a mild disease in adult cattle characterized by reduced milk production, pyrexia and diarrhea [4]. However, SBV infection of susceptible pregnant animals can be associated with musculoskeletal and central nervous system malformations in stillborn or newborn lambs and calves [3]. SBV was detected for the first time in November 2011 in plasma samples collected from cows displaying fever and diarrhea and farmed near the town of Schmallenberg, Germany [3]. The first acute infections associated with SBV were reported in August 2011, while the first malformations in stillborn animals caused by this virus were detected in The Netherlands in December 2011 [5]. Since then, 9 countries have reported congenital malformations and stillbirth associated with the presence of SBV as of May 2012 [6]. In some areas, SBV cross-reacting antibodies have been detected in as high as to 100% of the cattle surveyed [7], [8], although the clinical and consequent economic impact of this infection is not completely clear as yet [9].

Phylogenetic analysis revealed that SBV belongs to the genus *Orthobunyavirus* within the *Bunyaviridae*, a large family comprising hundreds of viruses able to infect a broad range of vertebrate and invertebrate hosts. Bunyaviruses are significant pathogens both in humans and animals and cause a range of diseases including febrile illnesses (Oropouche virus), encephalitis (La Crosse virus) and hemorrhagic fevers (Rift Valley fever virus) [10].

SBV clusters with viruses from the Simbu serogroup, in particular with viruses of the species *Sathuperi virus* including Sathuperi virus (SATV), Douglas virus (DOUV) and Shamonda virus (SHAV) currently classified within the *Shamonda virus* species [11]. Viruses from Simbu serogroup have been associated with abortions, stillbirths and malformations (arthrogryposis - hydranencephaly syndrome) in ruminants in Asia, Africa and Oceania. Akabane virus (AKAV) is the most widely studied member of the Simbu serogroup [12]–[17]. In the literature, there is relatively little information on the diseases (if any) associated with SATV and SHAV infection. Viruses from the Simbu serogroup have not been detected in Europe before and given that SBV has not been found in archived samples so far, it is likely that the first introduction of SBV in Europe occurred in spring 2011. Given the current available data, it is not possible to ascertain when and how SBV was introduced in Europe.

With the exception of hantaviruses, all Bunyaviruses are transmitted by arthropod vectors [10]. SBV is also thought to be an arbovirus given its close relatedness to viruses of the Simbu serogroup (all known to be transmitted by insects). In addition, SBV has also been detected in pools of *Culicoides* biting midges in Denmark [18].

Bunyaviruses are enveloped viruses and have a segmented single stranded RNA genome of negative or ambisense polarity. The viral genome comprises three RNA segments referred to as small (S), medium (M) and large (L) which encode four structural proteins: the nucleocapsid protein (N); two glycoproteins (Gn and Gc); and the viral polymerase. Members of the *Orthobunyavirus* encode two additional non-structural proteins, NSs and NSm. NSs is encoded by an open reading frame in the S segment overlapping the N gene while NSm is encoded by the M segment as a polyprotein that is co-translationally cleaved into NSm, Gn and Gc [10]. The NSs protein of Bunyamwera virus (BUNV), the prototype of the family, is a non-essential gene product for viral replication that has been shown to play a role in the inhibition of the host cell mRNA and protein synthesis. The NSs protein contributes to viral pathogenesis by blocking the production of interferon (IFN) through transcriptional inhibition and consequently inhibiting the innate responses of the host [19]–[21].

Studies on the molecular biology of RNA viruses has been transformed by the development of reverse genetics that allow the rescue of infectious virus from cloned cDNA copies of the viral genomes [22]. A reverse genetics for BUNV has proven invaluable in unraveling the underpinning mechanisms of viral replication and pathogenesis of this virus family [19], [21], [23], [24].

In this study, we developed reverse genetics platforms for SBV and used them to characterize the biology and pathogenesis of this emerging pathogen. We obtained SBV mutants revealing key determinants of viral virulence and show that the viral non-structural protein NSs counteracts the innate immunity of the host. In addition, we show that SBV replicates in neurons of both experimentally infected mice and in naturally occurring SBV infected lambs and calves.

## Results

### *In vitro* tropism of SBV

**Figure 1:**
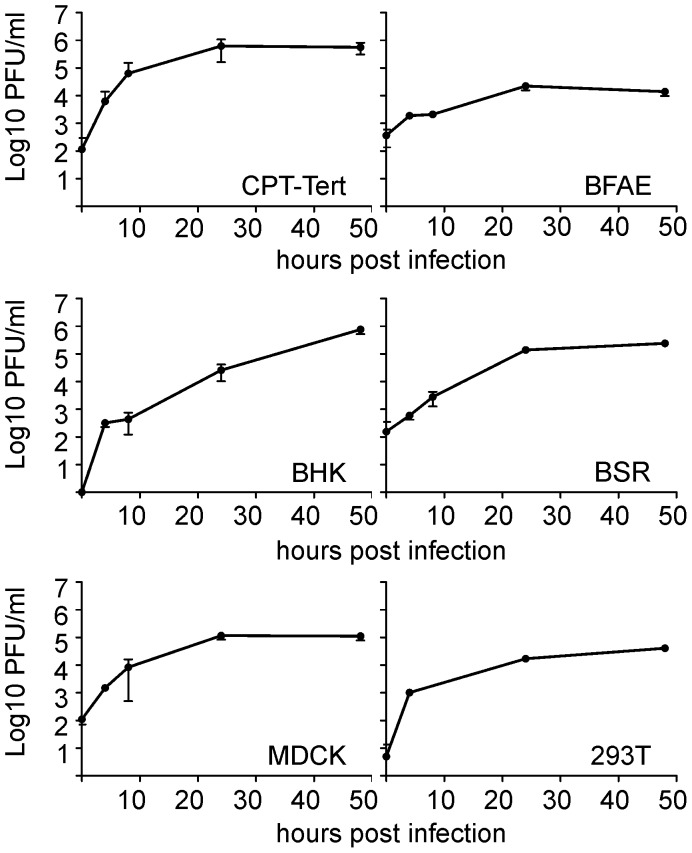
*In vitro g*rowth kinetics of SBV. A. Growth kinetics of SBV in a variety of established cell lines as indicated in the panel. Cells were infected with SBV at a MOI of 0.05 and supernatants were collected at 4, 8, 24 and 48 h post-infection. Supernatants were then titrated in CPT-Tert cells by limiting dilution analysis.

Our first goal was to gain insight into the *in vitro* growth kinetics of SBV in several cell lines derived from various animal species and humans. SBV grew efficiently in all the cell lines tested including sheep CPT-Tert, bovine BFAE, human 293T, dog MDCK and hamster BHK-21 and BSR cells (Figure 1). SBV reached titers of 10^6^ PFU/ml at 48 h post-infection and induced cytopathic effect (CPE) in most cell lines with the exception of BFAE (data not shown). We found SBV to replicate very efficiently in sheep CPT-Tert, where it induced well-defined plaques of approximately 3 mm in diameter at 72 h post-infection. Consequently, we used CPT-Tert for all the titrations of SBV performed in this study.

### Development of reverse genetics protocols for SBV

**Figure 2:**
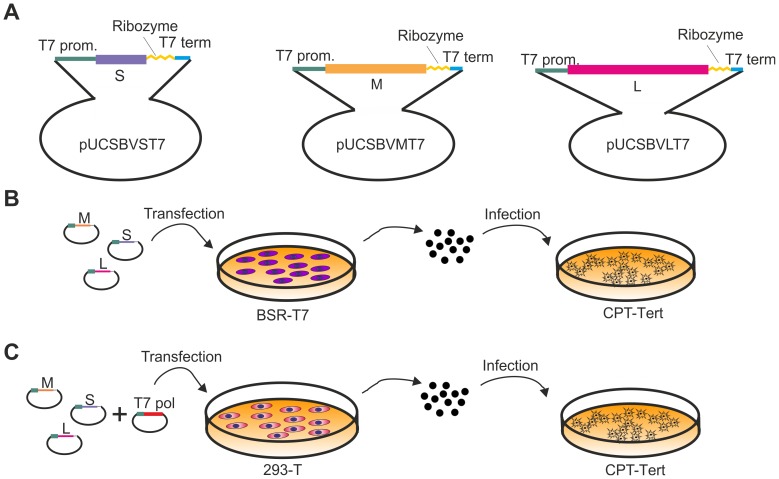
Reverse genetics protocols for the rescue of SBV. A. Schematic representation of the antigenome plasmids used to rescue SBV. B and C. Schematic representation of the strategies used for the rescue of SBV in BSR-T7/5 and 293T cells as described in the text. The two methods are very similar with the exception that BSR-T7/5 stably express the T7 RNA polymerase and therefore these cells are transfected only with the SBV antigenome plasmids. On the other hand 293T cells are transfected with the antigenome plasmids and an expression plasmid for the T7 RNA polymerase.

Several members of the *Bunyaviridae* have been successfully rescued by reverse genetics [25]–[28]. The most successful and simple approach involves the transfection of plasmids encoding full-length antigenome viral RNAs under the control of the T7 polymerase promoter. We adopted a synthetic biology approach and obtained the 3 plasmids harboring the full full-length antigenome RNAs of each viral segment by *in vitro* synthesis, using the sequences of the first SBV isolate available in the International Nucleotide Sequence Database Collaboration in January 2011 (accession numbers HE649912–HE649914) [3]. The complete sequences of the 5′ and 3′ untranslated regions (UTRs) of the SBV S, M and L segments were unavailable at that time and therefore we predicted the missing sequences based on the high similarity with the sequences of AKAV. The rescue plasmids were designed to contain the full-length antigenome RNAs flanked by the T7 promoter immediately upstream of the 5′ UTR and the hepatitis δ ribozyme followed by T7 terminator sequences immediately downstream of the 3′ UTR as previously described [29] within the pUC57 vector (Figure 2A). We transfected the 3 antigenome-encoding plasmids in BSR-T7/5 cells, a BHK-derived cell line that constitutively expresses the T7 RNA polymerase [30]. Five days post transfection supernatants were collected and the presence of virus was assessed by standard plaque assays in CPT-Tert cells (Figure 2B). Negative controls included cells transfected with only two of the rescue plasmids. We successfully rescued SBV following this method, although in some cases transfections did not result in viable rescued SBV.

**Figure 3:**
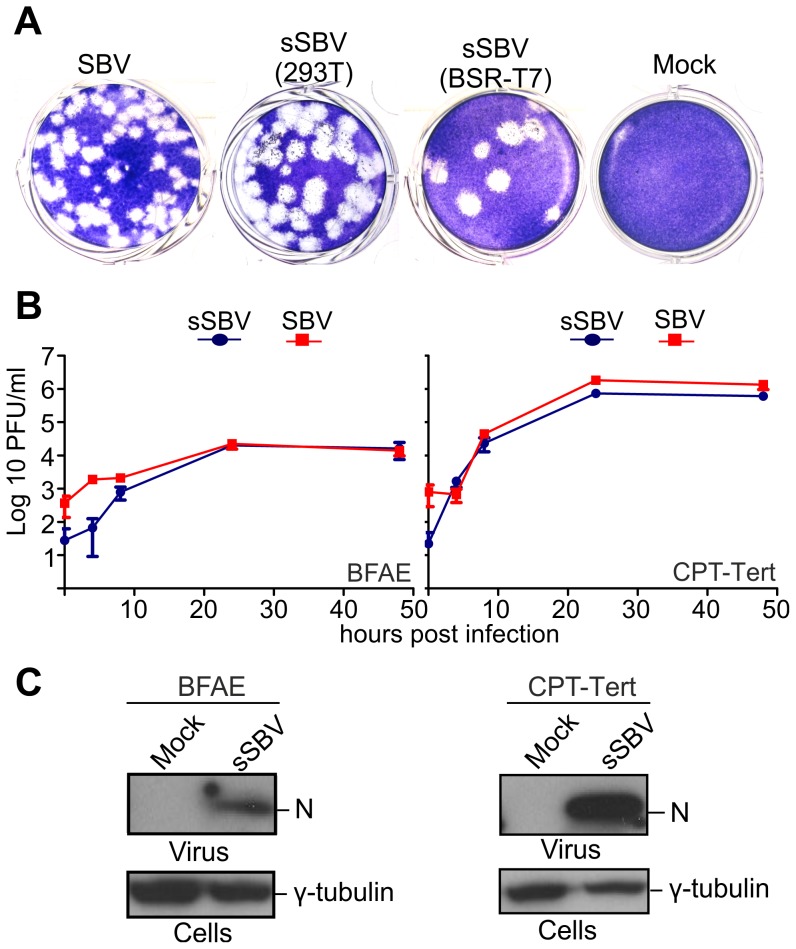
*In vitro* phenotypic characterization of SBV and sSBV. A. Comparison of plaques produced by wild type SBV and sSBV rescued using BSR-T7/5 and 293T cells. B. Growth kinetics of SBV and sSBV. CPT-Tert and BFAE cells were infected at a MOI of 0.05 for 90 min. Supernatants were collected at the indicated times post-infection and virus titer was measured using standard plaque assays in CPT-Tert cells. C. The presence of SBV in CPT-Tert and BFAE cells infected with wild type and rescued virus was confirmed by western blotting using antibodies against the SBV N (nucleocapsid) protein.

From the *in vitro* growth assays described above, it appeared that SBV infected 293T cells readily. Thus, we reasoned that it would be possible to rescue SBV more efficiently in this cell line known to be highly transfectable if we provided the T7 RNA polymerase *in trans*. To this end, we transfected 293T cells with the 3 SBV antigenome plasmids (pUCSBVST7, pUCSBVMT7 and pUCSBVLT7) along with a plasmid expressing the T7 RNA polymerase under the control of the CMV immediate early promoter (pCMV-T7) (Figure 2C). SBV was efficiently rescued following this method, which we found overall more reproducible. We were also able to rescue BUNV [31] in transiently transfected 293T cells (data not shown) demonstrating that this method can be easily applied to other viruses within this family. sSBV rescued in either 293T or BSR-T7/5 cells produced plaques of similar size and shape to SBV (Figure 3A). In addition, we found that sSBV grew with the same kinetics and reached approximately the same titers of SBV in both sheep CPT-Tert and bovine BFAE cells (Figure 3B). The presence of SBV in infected cells was confirmed by western blotting of cell lysates of infected cells (Figure 3C). These results indicate that wild type SBV and sSBV display similar phenotypic characteristics *in vitro*.

### Determination of the 3′ and 5′ UTR sequences of the SBV genome segments

**Figure 4:**
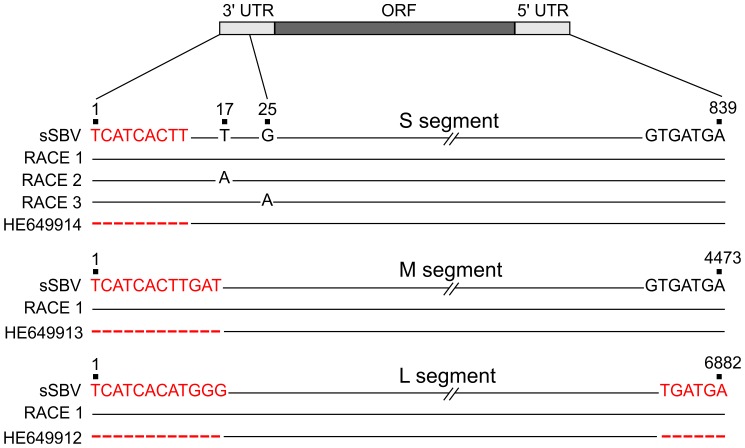
Sequences of 3′ and 5′ UTRs of SBV obtained by RACE PCR. A schematic representation of the genomes of the 3 viral segments is shown. Sequences on the top of each segment (sSBV) indicate the sequence of the UTRs of the plasmids used for reverse genetics. The middle sequences indicate the UTRs sequences inferred by RACE PCR (RACE1, RACE 2 etc). The bottom sequences indicate SBV UTRs reported in GenBank (HE649914, HE649913 and HE649912). Positions highlighted in red correspond to nucleotides inferred for the construction of sSBV sequences based on the AKAV sequence. Scores represent positions in the genome segments for which there was no sequence available in the submitted GenBank sequences. Note that 54 nucleotides previously reported in the 5′ UTR of the M segment that appeared to be a sequence artifact (i.e. not part of the SBV genome) were not, as expected, detected by RACE and are not shown in the figure. Numbers shown in this figure take into account the corrected 3′ and 5′ UTRs.

As mentioned above, we predicted the complete sequences of the 5′ and 3′ UTRs of the S, M and L segments of SBV on the basis of published AKAV sequences. We performed 5′ and 3′ RACE PCRs on RNA extracted from SBV-infected cells in order to establish that the sequence of sSBV faithfully represented the wild type field strain. The sequences of the 3′ and 5′ UTR of the M and L segments were 100% identical to the predicted sequences used in the rescue plasmids for sSBV (Figure 4). We did not find with our RACE PCR an extra 54 nucleotides that were reported in the 5′ UTR of the M segment (accession number HE649913) (not shown) which represent most likely a sequencing artifact beyond the correct 3′ end of the published antigenome sequence. We found polymorphisms in SBV at positions 17 and 25 of the 3′ UTR of the S segment. Some of the clones analyzed contained thymine (T) at position 17 while others contained an adenine (A) at that position. Similarly, at position 25, some clones harbored a guanine (G) while other clones contained an A. The 3′ UTR sequences of the synthesized plasmids used for reverse genetics contained a T at position 17 and a G at position 25 indicating that the original sequence prediction was correct (Figure 4). We also sequenced the remaining full genome of the SBV strain used in this study and we found it to be identical to the original sequences submitted in GenBank (data not shown).

### Characterization of SBV virulence in mice

**Figure 5:**
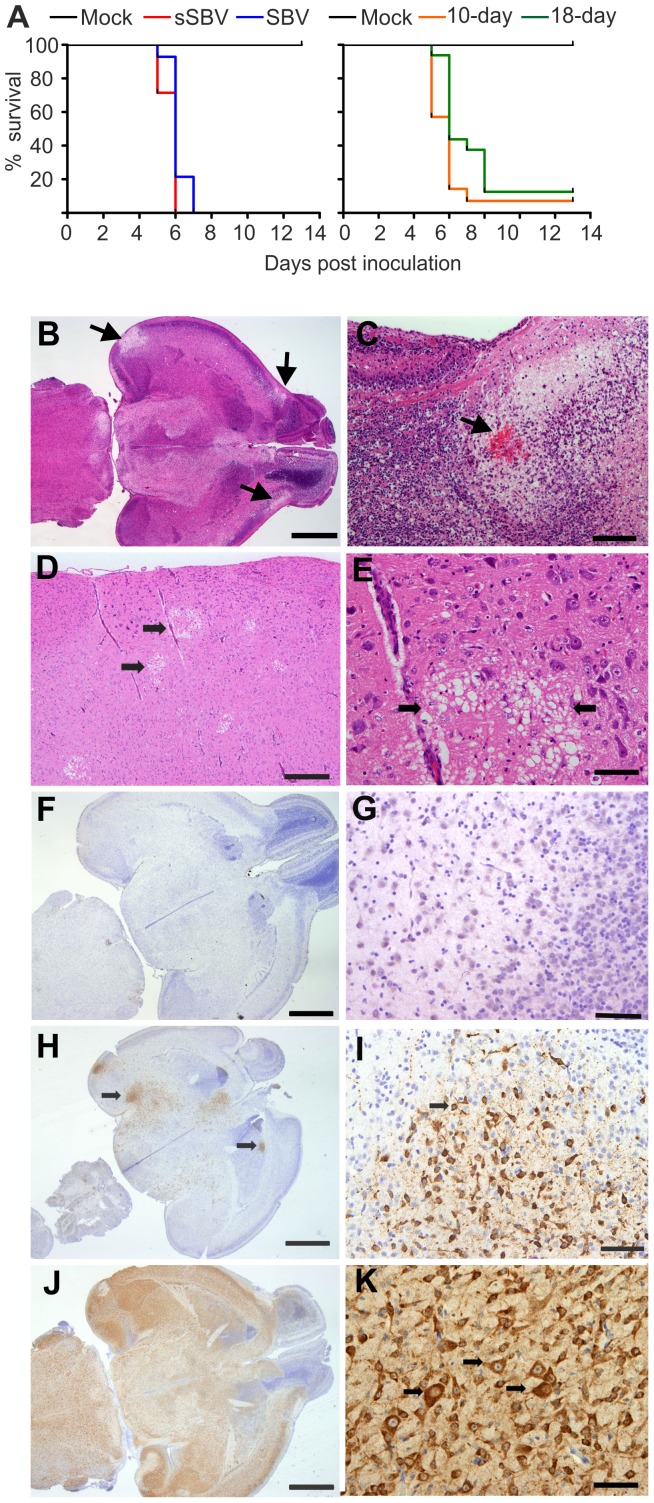
Neuropathology of SBV infection in mice inoculated intracerebrally. A. Left panel: survival plots of 2-day old NIH-Swiss mice inoculated intracerebrally using the indicated viruses or cell culture media as a control. Right panel: survival plots of 10 and 18-day old NIH-Swiss mice inoculated as described in A. B–E histopathology of brain sections from SBV-infected infected mice stained with hematoxylin. B and C. brain sections from SBV-infected mice at 72 h post-infection. Arrows indicate areas of malacia and hemorrhage. D and E brain sections from SBV infected mice at 120 h post-infection, showing areas of vacuolation in low and high magnification respectively. F–K immunohistochemistry of brain sections of mock-infected mice (F–G), or SBV infected mice at 48 h (H–I) or 72 h (J–K) post-infection using an SBV N antiserum as described in Materials and Methods. Bars = 2 mm in B, F, H, J; 200 µm in C; 500 µm in D; 100 µm in E, G, I, K.

We next investigated whether mice could be used as an experimental model of SBV infection and pathogenesis. Firstly, we inoculated 3 litters of 2-day old newborn NIH-Swiss mice (n = 8–14) intracerebrally with 400 PFU of SBV, sSBV or cell culture media as a control (study 1). All mice inoculated with SBV and sSBV died within 8 days post-inoculation while all control mice survived until the end of the experiment (Figure 5A, left panel). These data clearly indicate that sSBV is as virulent as SBV at least in this experimental model. In order to determine whether age susceptibility existed for SBV infection in this mouse model, we inoculated litters of 10 and 18-day old NIH-Swiss mice as described above with sSBV (study 2). We found that SBV infection was lethal for most of the infected mice of these age groups, displaying similar kinetics to the one displayed by SBV infection in newborn mice (Figure 5A, right panel).

Histopathology of brains collected at 72 h post-infection revealed bilateral symmetrical vacuolation and loosening of the neuropil of the superficial cerebral cortex and the mesencephalon (Figure 5B–C). In particular, we found small areas of haemorrhage within large areas of malacia (necrosis of brain tissue) in the cerebral cortex (Figure 5C, arrow). In brains collected at 120 h post-infection (Figure 5D–E) there was random multifocal vacuolation of the white matter of the cerebrum with small amount of nuclear debris (Figure 5E). There was a minimal, multifocal perivascular infiltrate of lymphocytes in the adjacent grey matter. The presence of SBV was confirmed by immunohistochemistry using a polyclonal antibody against the N protein. We found no reactivity in mock-infected mice (Figure 5F–G). We found patchy areas of positive staining in brain sections taken from mice euthanized 48 h post infection corresponding to the cytoplasm of neurons (Figure 5H–I). Extensive immunoreactivity for SBV was noted within the cerebrum and mesencephalon of mice euthanized 72 h after inoculation corresponding to infection of neurons. SBV antigens were also detected in cells that morphologically resembled astrocytes (Fig. 5 J–K). However, by immunofluorescence we were not able to detect SBV in cells expressing GFAP (glial fibrillary acidic protein), a specific marker for astrocytes (data not shown).

### SBV is neurotropic in naturally *in utero* infected lambs and calves

**Figure 6:**
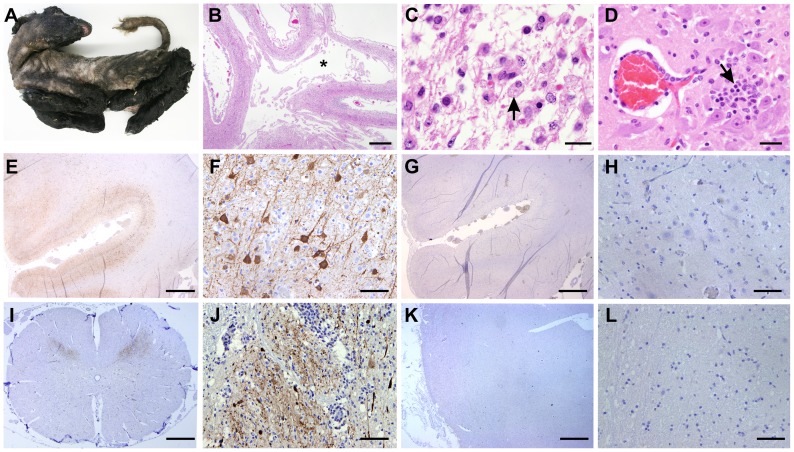
SBV tropism in the central nervous system of naturally *in utero* infected lambs and calves. A. Stillborn lamb showing signs of congenital SBV infection including artrogryposis, brachygnatia inferior and torticollis. B–D: Tissue sections stained with hematoxylin and eosin derived from brain of a lamb congenitally infected with SBV. Panel B illustrates cerebral cortex with porencephaly. The white matter is lacking as indicated by (*). Adjacent grey matter is reduced in thickness (bar = 2 mm). C. Higher magnification micrographs showing areas of malacia and the presence of myelin laden macrophages (arrow; bar = 20 µm). D. Glial nodule (arrow) and mild lymphohistiocytic perivascular infiltrate (bar = 50 µm). E–L: Immunohistochemistry of tissue sections derived from brain (E–H) or spinal cord (I–J) of lambs congenitally infected with SBV. In sections shown in panels E, F, I and J a SBV N polyclonal rabbit antiserum was used, while panels G–H show instead serial sections (of E and F) incubated with the pre-immune serum. The use of SBV N antiserum reveals a strong positive reaction (characterized by the intracytoplasmic dark brown staining) in the cell body and processes of neurons of the grey matter, while no staining was observed with serial sections incubated with the pre-immune serum. K–L: Immunohistochemistry of control tissue sections derived from brains of sheep reared in Scotland and probed with the antiserum towards the SBV N protein showed no immunoreactive cells.

Next, we analysed tissue sections of brain and spinal cord from a total of 8 naturally infected lambs and calves presenting malformations commonly shown by animals congenitally infected with SBV such as, arthrogryposis, brachygnatia inferior, torticollis and curvature of the spine (Figure 6A) [32]. All samples derived from abortion or stillborn cases that occurred in farms in an SBV-endemic area in Germany, although we cannot ascertain the time of infection nor the specific SBV strain involved in these cases. Histopathology of brain sections showed lesions commonly observed in SBV infected animals, including porencephaly associated with widespread tissue destruction (Figure 6B). Clusters of myelin laden macrophages (Fig. 6C, arrow) were noted in areas of rarefaction. Glial nodules were randomly scattered throughout the cerebrum (Fig. 6D, arrow) and there was a mild, multifocal perivascular infiltrate of lymphocytes and macrophages/microglia.

By immunohistochemistry, we detected SBV predominantly in the cell body and processes of neurons of the grey matter, similarly to what we have observed in mice (Figure 6E–F). In addition, we found expression of abundant SBV antigen also in the grey matter of the spinal cord (Figure 6I–J). Our controls included serial sections of brains collected from SBV-infected animals incubated with the pre-immune serum collected from the rabbit used for the production of the polyclonal antibody against SBV N protein (Figure 6G–H). No positive reaction was observed in all the SBV positive or suspected cases that we tested in this study. In addition, we used also as negative controls tissue sections collected from the brains of lambs or calves that died as result of unrelated pathologies from Germany (collected before 2001) or Scotland (n = 7). As expected, none of these negative controls showed immunoreactive cells (Figure 6K–L). Moreover, our studies strongly indicate that SBV replicates in neurones of brain and spinal cord of animal naturally infected with SBV.

### Molecular determinants of SBV virulence

**Figure 7:**
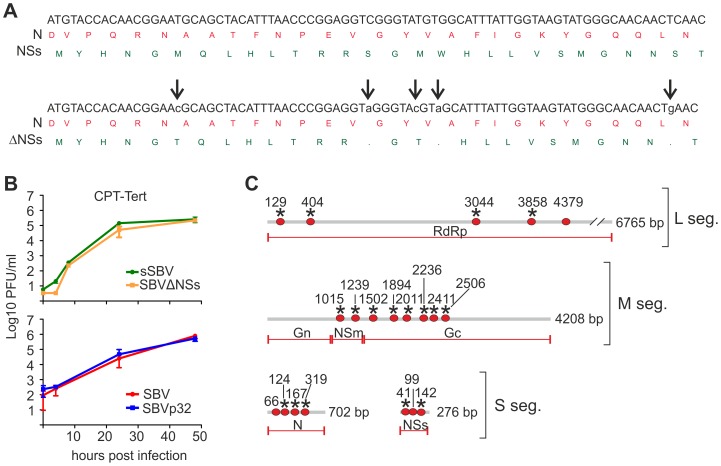
*In vitro* phenotypic characteristics of SBVΔNSs and SBVp32. A. Strategy used to generate SBVSΔNSs. Nucleotide (antigenome) and amino acid sequences showing the translation start site of the NSs in an overlapping reading frame within the N gene are indicated (top sequences). Bottom sequences indicate silent mutations (low case) introduced in N that abrogate expression of the NSs protein. B. Growth kinetics of SBV lacking the NSs protein and SBVp32. CPT-Tert cells were infected at a MOI of 0.05 for 90 min. Supernatants were collected at the indicated times post-infection and the virus titer was measured using standard plaque assays in CPT-Tert cells. C. Schematic representation showing the nucleotide differences (red dots) found in SBVp32 in relation to SBV. Only the coding regions of the antigenomes of the 4 known genes are shown. Non-synonymous mutations are indicated with an asterisk.

The data obtained in calves and lambs suggested that newborn NIH-Swiss mice inoculated intracerebrally can be an useful experimental model of SBV infection. We attempted to further exploit this tool in order to identify determinants of SBV virulence using two different approaches. First, we attempted to attenuate SBV in tissue culture by passaging the virus serially in CPT-Tert cells, after which the resulting virus was plaque purified twice and a stock generated in the same cells reaching a total of 32 passages in tissue culture (virus referred to as SBVp32). In addition, given that the NSs proteins of several Bunyaviruses including BUNV [19], Rift Valley fever virus (RVFV) [33], [34], AKAV [26] and La Crosse virus (LCV) [35] have been shown to play key roles in viral replication and pathogenesis, we attempted to rescue by reverse genetics a SBV mutant lacking this non-structural protein. Similarly to other orthobunyavirus, the predicted NSs of SBV is encoded by the S segment in an overlapping reading frame within the N gene. Because of the inherent capabilities of RNA viruses to mutate and revert to a wild type phenotype, we introduced 2 silent mutations in pUCSBVST7 within the N gene, abrogating the second and third initiation codons of the NSs gene. Moreover, we introduced 3 premature stop codons within NSs that did not affect the N reading frame (Figure 7A). The resulting SBV NSs deletion mutant (termed SBVΔNSs) was then rescued in 293T cells as described above. We confirmed the identity of the deleted NSs mutant by sequencing.

We hypothesized that sequence changes were likely to have occurred in SBVp32 following extensive passage and plaque purification in tissue culture. Consequently, we amplified the coding regions of all 3 SBV genome segments by RT-PCR and sequenced the PCR products directly without cloning. We identified a total of 17 nucleotide changes among the 3 genome segments, most of which were non-synonymous mutations (Figure 7C). Most of the mutations were identified within the M and S segments. In particular, all the mutations found in the M segment were located within Gc and NSm proteins and none within Gn. We found that the S segment harbored the highest number of mutations in relation to its size. Three of the 4 changes found in the S segment of SBVp32 were non-synonymous mutations of the N protein. In addition, 3 of these mutations also affected the NSs protein leading to 1 synonymous and 2 non-synonymous changes.

We next characterized the *in vitro* growth properties of the rescued SBVΔNSs and SBVp32 in sheep cells (Figure 7B). We found that both SBVΔNSs and SBVp32 had comparable growth kinetics to sSBV and SBV respectively and both viruses reached similar titers in infected cells.

### Virulence of SBVΔNSs and SBVp32 in experimentally infected mice

**Figure 8:**
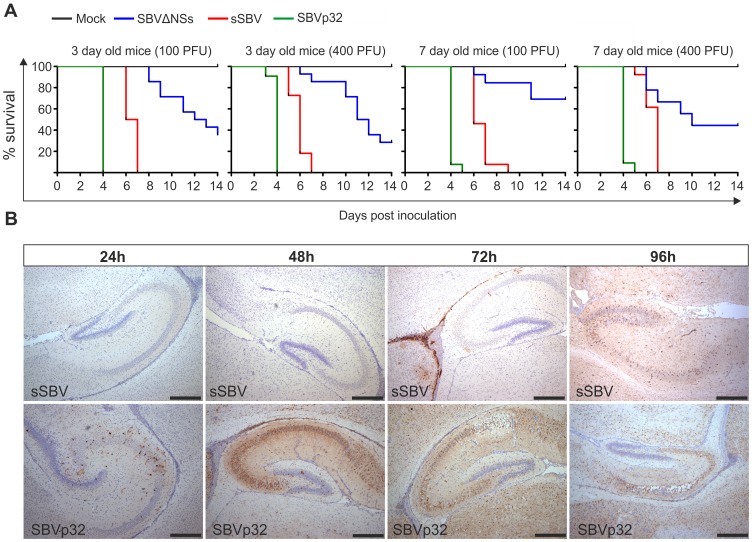
Virulence of SBV mutants in suckling NIH-Swiss mice. A. 3 and 7-day old mice were inoculated intracerebrally with either 100 or 400 PFU with the indicated viruses or cell culture media as a control. Survival plots show that SBVΔNSs possesses an attenuated phenotype while SBVp32 is more virulent than sSBV. B. Immunohistochemistry of brain sections derived from NIH-Swiss mice inoculated with sSBV or SBVp32 and killed at various time points post-infection as indicated in the figure. Immunohistochemistry was performed using an SBV N antiserum as described in Materials and Methods. At the early time points SBV antigens are detected only in sections derived from SBVp32 infected mice (Bar = 500 µm).

Thereafter, we inoculated litters of 3 and 7-day old NIH-Swiss mice intracerebrally with either 100 or 400 PFU of sSBV, SBVΔNSs, SBVp32 or cell culture media as a control (study 3). All mice inoculated with sSBV died within 7 days post-inoculation, with the exception of the litter of 7-day old mice inoculated with 100 PFU where 100% mortality was reached at day 9 post-inoculation (Figure 8A). All control mice were healthy until the end of the experiment. SBVΔNSs showed an attenuated phenotype. There was a clear delay in the time of death in the groups inoculated with SBVΔNSs and 40–60% of the inoculated mice survived infection. Unexpectedly, SVBp32 was more virulent than sSBV in this experimental model. Inoculation of SBVp32 resulted in 100% lethality by day 4 and 5 post-infection in 3 and 7-day old mice respectively (Figure 8A). To gain insight into the nature of the enhanced pathogenicity of SBVp32 observed in our mouse model of infection, we inoculated litters of 7-day old NIH-Swiss mice intracerebrally with SBVp32, sSBV (and media as a control) and brain samples were collected at different time points post-infection (study 4). Brain sections were analyzed by immunohistochemistry to detect the presence of SBV. We detected the presence of foci of virus-infected cells as early as 24 h post-infection in samples derived from mice infected with SBVp32 (Figure 8B). In these mice, SBV antigens were widely spread by 48 h post-infection. In contrast, we first detected virus antigens at 72 h post-infection in brain sections derived from mice infected with sSBV. These results suggest that SBVp32 is able to spread faster than sSBV in the brain of experimentally infected mice.

### The NSs protein of SBV counteracts the IFN response of the host

**Figure 9:**
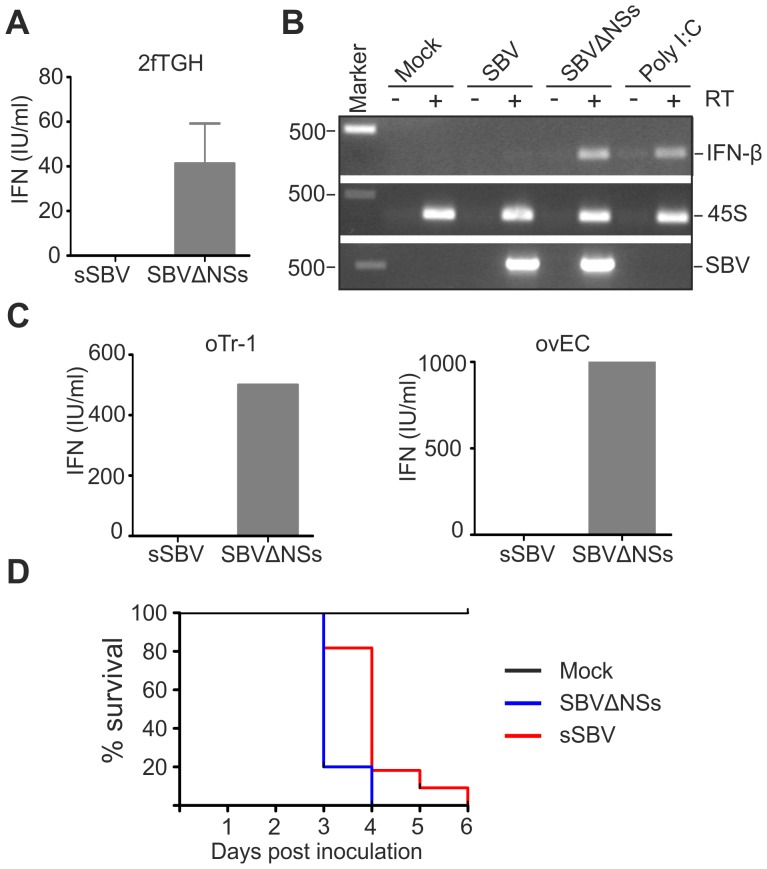
SBVSΔNSs induces the production of IFN. A. IFN protection assay. 2fGTH cells were infected with the indicated viruses and supernatants were collected 24 h post infection. Supernatants were UV treated to remove infectious virus and fed to CPT-Tert cells after serial dilution. CPT-Tert cells were later infected with EMCV and the presence of CPE monitored and compared to cells supplemented with known amounts of universal IFN. B. The induction of IFN-β mRNA was investigated by RT-PCR from RNA extracted from 2fGTH cells infected with virus as indicated or transfected with Poly I∶C as a positive control. To control for the presence of residual genomic DNA all the samples were amplified after reverse transcription performed without reverse transcriptase (top panel, indicated as+/−RT). The quality of the extracted RNA was verified by the amplification of the 45S ribosomal RNA (middle panel). The presence of virus was confirmed by the amplification of part of the SBV S segment (bottom panel). C. IFN protection assay performed in primary ovine trophoblast cell (oTr-1) and primary ovine endothelial cells as described in A. D. Survival plots of 7 day old IFNAR^(−/−)^ mice inoculated intracerebrally with sSBV, SBVSΔNSs or cell culture media as a control. Data indicate that SBVΔNSs is as virulent as sSBV in these mice that lack an intact IFN system.

The attenuation of virulence displayed by SBVΔNSs suggested that the NSs protein plays an important role in viral pathogenesis. The NSs proteins of Orthobunyaviruses have been shown to indirectly inhibit synthesis of IFN-α and β by shutting down cellular mRNA synthesis [20], [22], [28], [36]. Consequently, we performed a series of assays to determine whether SBV and SBVΔNSs induce the synthesis of IFN in infected cells. Firstly, we tested the human 2fTGH cells, which are known to be IFN competent. Cell lines were infected with sSBV or SBVΔNSs and at 24 h post-infection the supernatant were collected and virus UV inactivated. An IFN-protection assay was then performed in CPT-Tert cells, which respond to IFN but are defective for its production (data not shown) and thus are a good target cell line for these experiments. Virus-inactivated supernatant from 2fTGH cells was added to CPT-Tert cells and incubated for 24 h before infection with encephalomyocarditis virus (EMCV), a virus susceptible to IFN [37]. In parallel, cells were also incubated with a known amount of universal IFN as a control. We found that supernatants from cells infected with sSBV did not contain IFN as they were not able to protect CPT-Tert cells from infection with EMCV (Figure 9A). In contrast, supernatant from cells infected with SBVΔNSs was able to confer protection against infection of the target cells by EMCV, and contained an average of 40 IFN international units per ml (IU/ml).

Secondly, we investigated whether the lack of IFN production upon infection with sSBV was due to the inhibition of transcription of the type I IFN gene. To this end, we investigated the presence of IFN-β mRNA by RT-PCR in 2fTGH cells following infection with sSBV and SBVΔNSs, or transfection with poly I∶C as a positive control. We did not detect IFN-β mRNA in sSBV-infected or mock-infected cells, while it was clearly detectable in cells infected with SBVΔNSs or transfected with poly I∶C (Figure 9B). These results suggest that the SBV NSs protein interferes with transcription of the IFN-β gene upon infection.

Furthermore, in an attempt to evaluate the data obtained above in a more relevant cell culture system, we repeated the IFN protection assays in sheep primary endothelial cell cultures (ovEC) and ovine trophoblast cells (oTr-1). Similarly to the data obtained in human cells, we found that the supernatant from sSBV-infected cells did not protect CPT-Tert cells from infection with EMCV. On the other hand, supernatant from SBVΔNSs-infected ovEC and oTr-1 was found to contain abundant amounts of IFN (estimated to be between 500 and 1000 IU/ml; Figure 9C).

Finally, to evaluate the role of SBV NSs protein in counteracting the IFN response of the host *in vivo*, we inoculated litters of 7 day old IFN receptor null mice (IFNAR^(−/−)^) intracerebrally with sSBV (n = 11), SBVΔNSs (n = 10) and culture media as a control (n = 8) (study 5). All the control mice survived during the observation period (Figure 9D). All mice inoculated with SBVΔNSs died by 4 post-infection mice while 80% of mice infected with sSBV died at the same time point and the remaining 20% died by day 6 post-infection. These data strongly suggest that animals lacking a competent IFN response are equally susceptible to sSBV and SBVΔNSs, confirming the role of the NSs protein as a modulator, at least indirectly, of the IFN response *in vivo*.

## Discussion

SBV is a new emerging orthobunyavirus that has been associated with abortions and malformations in sheep and cattle. The *Bunyaviridae* include many viruses that have emerged or re-emerged in the last decade such as OROPV, Henan fever virus, Crimean-Congo hemorrhagic fever, RVFV, LCV and others [38]–[42]. SBV was isolated for the first time in Germany in October 2011 [3]. However, the virus probably entered Northwestern Europe in the border region of The Netherlands, Germany and Belgium [8] and subsequently spread rapidly to neighboring countries, including Luxembourg, France, Italy, Spain, Denmark and the United Kingdom. In this study, we have established an experimental platform that comprises both *in vitro* and *in vivo* systems to study SBV biology and pathogenesis.

Studies on SBV have thus far been concentrated in understanding the animal species susceptible to this virus, the spreading of the infection, and its impact on animal health and the farming industry. In this study, we undertook several steps towards understanding the molecular biology of SBV, its cellular tropism, pathogenesis and host-virus interaction. Using a synthetic biology approach, we developed two reverse genetics protocols for SBV, as powerful tools to characterize this emerging pathogen and to manipulate its genome. Using SBV sequences deposited in public databases, we synthesized the three SBV antigenome segments *in vitro* and rescued replication competent SBV in BSR-T7/5 transfected cells, as previously described for other bunyaviruses [29]. We also developed an alternative rescue system, using the plasmids above in transiently transfected 293T cells, where the T7 RNA polymerase was provided *in trans*. Using RACE RT-PCR performed on viral RNA extracted from early passages of SBV stocks we confirmed that the sequence identity of the viral UTRs in our synthetic plasmids indeed corresponded to SBV. This allowed us to provide for the first time the full-length sequence of circulating SBV since previous reports only provided partial sequences of the viral UTRs.

The “synthetic” SBV (sSBV) that we obtained was able to replicate *in vitro* as efficiently as wild type SBV. In addition, both wild type and sSBV were lethal in suckling mice injected intracerebrally. This experimental mouse system proved to be very useful in order to define SBV tropism. The histological lesions within the brains of mice inoculated with SBV show a progression from per-acute hemorrhage at 48 h post-infection to malacia at 72 h that extends to more widespread vacuolation of the white matter at 96–120 h post-inoculation. The strong immunoreactivity for SBV within the cerebral neurons of mice euthanized as early as 48 h post-inoculation, confirms that the histological changes within the brain are related to SBV infection. Importantly, we also found SBV to replicate in neurons of *in utero* infected calves and lambs. In fetal sheep, SBV infection can result in cavitation of the white matter of the cerebrum, cerebellar hypoplasia, mild lymphohistiocytic perivascular encephalitis, and small glial nodules scattered throughout the brain [32], [43]. Thus, the cavitary lesions observed in naturally SBV infected lambs/calves appear to be the natural progression of vacuolar changes within the white matter observed suckling mice acutely infected with SBV. The similarities in the pathologic changes between naturally infected fetal sheep and mice inoculated with SBV intracerebrally, suggest that the mouse can be a useful model to study at least some aspects of SBV virulence.

In this study we have made substantial progress by generating an SBV antiserum that allowed us to define that neurons in the brain seem to be the major target for viral replication in the developing fetus. The limited time frame in which sheep fetuses seem to be susceptible to SBV infection (28–50 days of gestation) [44] coincides with the development of the blood brain barrier (BBB). In sheep the BBB starts to develop between days 50 and 60 of gestation and reaches full development by day 123 [45]. Thus, the virus could have easy access to the brain of the fetus during a short period of time: from day 28 of gestation when the placentomes (functional units of exchange between mother and fetus in the ruminant placenta) develop until day 50 when the BBB starts to develop. This would explain why disease in adult animals, that have an intact BBB, is mild with no apparent development of lesions in the CNS. Newborn mice have a fully mature BBB, however the developing cerebral vessels can be fragile and allow leakage of infectious agent [46]. SBV could potentially reach the mouse brain tissue from subcutaneous inoculation proving an opportunity to develop a challenge model of infection that would better mimic natural exposure. It has been reported that subcutaneous inoculation of IFNAR^−/−^ mice with SBV resulted in weight loss and 10–20% mortality, therefore a different aspect of SBV infection could be explored also with this model [47].

The malformations and deformities observed in SBV-infected lambs and calves are accompanied by muscle hypoplasia and demyelination. Here, we found SBV to infect the neurons of the grey matter of the spinal cord, which would suggest that muscular hypoplasia and muscular defects observed in SBV infected lambs and calves are mostly secondary to damage of the central nervous system (CNS).

Using reverse genetics we engineered a SBV NSs deletion mutant as this protein has been found in other orthobunyaviruses to be a virulence factor, acting by inhibiting cellular transcription and therefore indirectly antagonizing the host IFN response. SBVΔNSs replicated at levels comparable to wild type SBV in established cell lines, indicating that the NSs protein is not essential for viral replication *in vitro*. However SBVΔNSs was strongly attenuated in newborn mice. It is unlikely that the attenuation observed for SBVΔNSs is due to a change in cell tropism. On the contrary, we attribute its attenuated phenotype to its inability to counteract the innate immune response of host. This is supported by the fact that we were able to detect induction of IFN in IFN-competent human cell lines and in sheep primary cells when they were infected with SBVΔNSs but not when they were infected with wild type SBV. The production of IFN in SBVΔNSs-infected cells was associated with the presence of IFN-β transcripts, which were not detected in cells infected with wild type SBV indicating that the inhibition of IFN production occurred at the level of transcription. Most importantly, we found no differences in the virulence of sSBV and SBVΔNSs in IFNAR^(−/−)^ mice, further reinforcing the notion that the SBV NSs protein is able to modulate at least indirectly the host innate immune response. Thus, the NSs protein of SBV acts as a virulence factor. Similar roles have been identified for the NSs proteins of RVFV [36] and LCV [35] underlying the importance of overcoming the host innate immune response for efficient viral replication.

In an attempt to identify other determinants of SBV virulence, we serially passaged SBV in CPT-Tert cells. Normally, serial passage of pathogenic viruses in cell culture results in decreased virulence especially when the cells used are defective in the production of factors mediating innate immune responses. However, using this approach, we unexpectedly obtained an SBV mutant (SBVp32) with increased pathogenicity in suckling mice. SBVp32 appeared to spread more rapidly than SBV in the brain of infected animals. SBVp32 accumulated a variety of mutations in all 3 viral segments. Of these, the ones found in the M and S segment are potentially the most interesting ones given that: i) we have already shown that the NSs is a virulence factor; and ii) most of the mutations found in the M segment are present in the Gc protein that is exposed in the outer surface of the virion and thus is the principal target of neutralizing antibodies. The mutations generated in Gc in an immunological unconstrained environment could be associated for example with an increased cell receptor affinity.

In conclusion, our synthetic biology approach demonstrates that, at least for the *Bunyaviridae*, it is possible very rapidly to investigate and characterize an emerging virus whilst only having knowledge of the complete sequence of the virus. Using synthetic plasmids we developed 2 reverse genetic protocols for the rescue of replication competent SBV. Most importantly, we developed a mouse model of infection that allowed us to identify a viral gene critical for virulence. Viral tropism for neurons of the CNS was shown to be similar in experimentally infected mice and naturally infected calves and lambs. Altogether, the molecular virology tools that we generated and the findings of this work open new avenues to study the biology and pathogenesis of a novel and rapidly spreading emerging virus.

## Materials and Methods

### Ethical statement

All experimental procedures carried out in this study were approved by the ethical committee of the Istituto G. Caporale (protocol number 5383/2012) and further approved by the Italian Ministry of Health (Ministero della Salute) in accordance with Council Directive 86/609/EEC of the European Union and the Italian D.Igs 116/92.

### Cell lines

The 293T, 2fTGH, BHK-21, BSR and MDCK cell lines were grown in Dulbecco's modified Eagle's medium (DMEM) supplemented with 10% fetal bovine serum (FBS). BSR-T7/5 (provided by Karl Conzelmann) cells stably expressing the T7 polymerase were grown in Glasgow modified Eagle's medium supplemented with 10% FBS, 10% of tryptose phosphate broth and G418 at a final concentration of 1 mg/ml. Sheep choroid plexus cells (CPT-Tert) were grown in Iscove's modified Dulbecco's medium supplemented with 10% FBS (provided by David Griffiths) [48]. Bovine fetal aorta endothelial cells (BFAE, Health Protection Agency collection number 87022601) were cultured in Ham's F12 medium supplemented with 20% FBS. Ovine trophoblast cells, oTr-1, (provided by Thomas Spencer) [49] were cultured in DMEM/F12 media supplemented with 15% FBS, 1 mM pyruvate, 700 nM of human recombinant insulin and 0.1 mM of nonessential amino acids. All cell lines were cultured at 37°C in a 5% CO_2_ and 95% humidified atmosphere and were supplemented with 10,000 U/ml of penicillin and 10 mg/ml of streptomycin.

### Primary endothelial cell cultures

Ovine aortic endothelial (ovEC) cells were isolated by collagenase treatment using a method adapted from Gillespie et al. [50]. Aortas were harvested from recently euthanized animals and washed twice in sterile PBS. The aortas were then incubated at room temperature for 1 h in DMEM supplemented with 5% FBS, 25 µg/ml penicillin/streptomycin and 50 ng/ml amphotericin B. After incubation, the aortas were placed into collagenase in a Petri dish (2 mg/ml in DMEM) for 1 h at 37°C. After incubation, the endothelial cells were removed by scraping and seeded in 6-well plates. Cells were maintained at 37°C and 5% CO_2_ in large vessel endothelial cell basal medium (TCS cellworks) supplemented with 20% FBS, human large vessel endothelial cell growth supplement (TCS cellworks), 25 µg/ml penicillin/streptomycin and 50 ng/ml amphotericin B. Cells were confirmed as endothelial cells by assessing their morphology using light microscopy and by immunofluorescence using antibodies against endothelial and smooth muscle cell markers. Cells used for this study were only passaged once.

### Antibodies

Antisera used in this study included a rabbit polyclonal antiserum against the SBV N protein expressed in bacteria as Glutathione-S-transferase (GST)-tagged recombinant protein (Proteintech). Antibodies against CD-31 (marker for endothelial cells) and actin (smooth muscle marker) were purchased from Source Bioscience and Abcam respectively.

### Viruses

Wild type SBV was originally isolated at the Friedrich-Loeffler-Institut (Germany). This virus was initially isolated from the blood of an infected cow and passaged once in KC cells and 6 times in BHK-21 cells. The virus was plaque purified and stocks were produced in BHK-21 cells. Virus titers were determined by standard plaque assays in CPT-Tert cells and expressed as plaque forming units per milliliter (PFU/ml). Encephalomyocarditis virus was provided by Rick Randall [37].

### Plasmids

pUCSBVST7, pUCSBVMT7 and pUCSBVLT7 encode the full-length antigenomic S, M and L SBV segments and were used for the SBV reverse genetics protocols described in this study (see below). In each plasmid, the SBV antigenome is placed downstream of the bacteriophage T7 promoter and upstream of the hepatitis δ ribozyme and the T7 terminator similarly to what described previously for other Bunyaviruses [29]. Sequences of pUCSBVST7, pUCSBVMT7 and pUCSBVLT7 were synthesized commercially and were derived from the incomplete SBV sequences available in GenBank in January 2012 (HE649912; HE649913; and HE649914). Where missing, the 5′ and 3′ end sequences for each genome segment (not available in GenBank at that time) were predicted by alignment of AKAV 5′ and 3′ sequences. pUCSBVΔNSsT7 was obtained by inserting 5 point mutations in pUCSBVST7 as described below. Two mutations altered the second and third initiation codons in the NSs while the remaining 3 introduced premature stop codons. pCMV-T7 expresses the T7 polymerase under the control of the CMV immediate early promoter.

### Reverse genetics

293T cells or BSR-T7/5 cells were plated in 6 well plates one day before transfection without in a final volume of 2 ml of tissue culture media without antibiotics. For rescue in BSR-T7/5 cells, 1 µg of each antigenome-encoding plasmid was transfected using Fugene HD at a 1∶3 ratio (µg of plasmid: µl of Fugene). For rescue in 293T cells, a plasmid expressing the T7 polymerase under the control of the CMV promoter was transfected alongside the plasmids containing the SBV antigenomes (or derived deletion mutant antigenomes). In both protocols, cells transfected with only two genomic segments were used as negative control. Supernatants were collected 5 days post transfection, clarified by low speed centrifugation and the presence of virus (termed sSBV for “synthetic SBV” in this study) was assessed by standard plaque assays in CPT-Tert cells as already described [51]. Virus stocks were generated from plaque purified virus in CPT-Tert cells.

### Virus growth curves

The *in vitro* growth kinetics of SBV was determined by infecting a variety of cell lines at a multiplicity of infection (MOI) of 0.05. The presence of infectious virus was then assessed from clarified supernatants collected at different time points post infection by end point dilution analysis performed in CPT-Tert cells. Each experiment was performed in triplicate and repeated at least twice.

### RACE analysis

The 5′ and 3′ ends of the 3 genome segments of SBV were sequenced using the 5′/3′ RACE kit according to the manufacturer's instructions (Roche). 5′ RACE was carried out on the genome strand to obtain the sequence of the 5′ end of the virus segments. Briefly, 1 µg total RNA extracted from SBV infected BHK-21 cells was reverse transcribed using a gene specific cDNA synthesis primer. The synthesised cDNA was column purified and poly-A tailed at the 3′ end and then amplified using the oligodT anchor primer and a second nested gene specific primer. To obtain the 3′ end of the virus segments, 3′ RACE was performed on the genome strand. Briefly, 1 µg total RNA extracted from SBV infected BHK-21 cells was poly-A tailed at the 3′ end of the RNA using poly A polymerase (NEB) according to the manufacturer's protocol. The poly-A tailed RNA was reversed transcribed using the oligodT anchor primer. cDNA was then amplified using the anchor primer and a nested gene specific primer. Primer sequences are available upon request.

### Interferon detection

2fTGH cells were plated at a density of 1.7×10^5^ cells/ml in 24 well plates. Cells were infected with the indicated viruses at MOI of 5 or transfected with poly I∶C (10 ng) using Lipofectamine 2000 according to the manufacturer's protocol. Cells were incubated at 37°C for 24 h, then lysed in TRizol Reagent (Invitrogen) and RNA extracted according to the manufacturer's protocol. 1 µg of total RNA was reverse transcribed using Superscript III (Invitrogen) according to the manufacturer's instructions. This cDNA was used as a template for amplification of the SBV S segment or 45S RNA using GoTaq polymerase (Promega) following the manufacturer's protocol. The sequences of the primers used for SBV detection are available on request. Primers for the amplification of the IFN-β and 45S RNAs have been previously published [52], [53].

### Interferon protection assays

Measurement of IFN levels was based on the methods described previously [37], [54]. 2fTGH, OvEC and oTr-1 cells were first seeded in 12-well plates and incubated for 2 days. Cells were then infected with the appropriate virus at a MOI of 5, 4 and 2 respectively and the medium was collected 24 h later. The medium was UV-treated for 5 minutes using a Spectrolinker XL-1500 UV crosslinker (Spectronics Corporation) in order to remove any infectious virus. CPT-Tert cells were then seeded in 96-well plates and 24 h later 2-fold serial dilutions (in duplicate) of the UV-treated media were added. Cells were then further incubated for 24 h. In parallel, cells were also incubated with 2-fold dilutions of a known amount of universal IFN. Cells were then infected with encephalomyocarditis virus (EMCV) and incubated for an additional 48 h. The levels of IFN were calculated by monitoring wells that were protected from cell death induced by EMCV and comparing them to the universal IFN control.

### Western blotting

SDS-PAGE and Western blots were performed from total cell lysates prepared 24 h post-infection as previously described [55], [56]. SBV was detected using a rabbit polyclonal antibody against the SBV nucleocapsid protein.

### *In vivo* pathogenicity studies

Animal experiments were carried out at the Istituto G. Caporale (Teramo, Italy) following local and national approved protocols for animal experimentation. **Study 1.** Litters of 2-day old NIH-Swiss mice were inoculated intra-cerebrally with 400 PFU of SBV, sSBV or mock inoculated using cell culture medium. Animals were monitored daily for signs of disease for a period of 14 days.

**Study 2.** Litters of 10-day and 18-day old NIH-Swiss mice were inoculated intracerebrally with 400 PFU of sSBV and monitored for signs of disease as above.

**Study 3.** Litters of 3 and 7-day old mice were inoculated intracerebrally with 100 or 400 PFU with sSBV, SBVp32, SBVΔNSs or cell culture media and monitored for signs of disease for a period of 14 days.

**Study 4.** Litters of 7-day old NIH-Swiss mice were inoculated intracerebrally with 400 PFU with sSBV, SBVp32, or cell culture media and 2 animals per group were euthanized at 24, 48, 72 and 96 h post-infection.

**Study 5.** Litters of 4 day old mice deficient of the type I IFN receptor (IFN Alpha Ro/o IFN^(−/−)^ 129/Sv) were inoculated intracerebrally with 400 PFU with sSBV, SBVΔNSs, or cell culture media as a control and monitored for signs of disease.

### Histopathology and immunohistochemistry

Brains from infected and control mice were collected and fixed in 10% neutral buffered formalin and paraffin embedded following standard histological procedures. In addition, brain tissues were also collected from 6 lambs and 5 calves born in North Rhine-Westphalia, a SBV-endemic area of Germany. At post-mortem, animals presented a variety of malformations including arthrogryposis, brachygnatia, torticollis, cerebellar hypoplasia, hydrocephalus, muscular hypoplasia. Negative controls included brain sections derived from a sheep and 3 calves, which died as a result of diseases unrelated to SBV-infection in Germany between 1994 and 2001. We also used as negative controls 3 brain tissues from Scotland, an area so far free of SBV infection. Tissue sections (4–6 µm) from mice, lambs and calves were stained with hematoxylin and eosin for histopathological examination. Sections were also examined for the presence of the SBV N protein using a specific antiserum and the EnVision (DAKO) detection system as described before [51], [57].

### Accession numbers

The sequences of the synthetic SBV genomic segments have been deposited in GenBank (accession numbers JX853179, JX853180, JX853181).
